# Case Report: Positive *Mycoplasma pneumoniae* IgM does not necessarily indicate acute infection: two case studies

**DOI:** 10.3389/fped.2025.1520021

**Published:** 2025-05-30

**Authors:** Hao Wang, Xiaoying Liu, Yabin Wu, Xin Cao, Jie Liu, Wei Li

**Affiliations:** ^1^ Maternal and Child Health Hospital of Hubei Province, Wuhan, China; ^2^ Hubei University of Chinese Medicine, Wuhan, China; ^3^ Hubei Provincial Hospital of Traditional Chinese Medicine, Wuhan, China; ^4^ Hubei Shizhen Laboratory, Wuhan, China; ^5^ Affiliated Hospital of Hubei University of Chinese Medicine, Wuhan, China

**Keywords:** *Mycoplasma pneumoniae*, pneumonia, immunoglobulin M, microbial infection, drug resistance, macrolides, case report

## Abstract

**Background:**

We aimed to explore the duration of IgM antibodies against *Mycoplasma pneumoniae*.

**Methods:**

Data from two children who consistently tested positive for *M. pneumoniae* IgM antibodies were retrospectively analyzed. Moreover, we examined the etiological data and drug use of these cases. Serologic testing using the colloidal gold method, direct chemiluminescence technique, and specific immune agglutination test were utilized. Quantitative PCR was used to detect *M. pneumoniae* in bronchoalveolar lavage fluid and antigen tests and nucleic acid detection were conducted for other respiratory pathogens.

**Results:**

The serological positivity of *M. pneumoniae* IgM antibody persisted for nearly ten months in one child and more than fifteen months in the other child. Furthermore, the persistently positive *M. pneumoniae* IgM antibody tests led to the inappropriate use of macrolides during multiple hospitalizations.

**Conclusions:**

IgM antibodies against *M. pneumoniae* may remain positive for an extended duration. Therefore, a positive *Mycoplasma pneumoniae* IgM test does not necessarily indicate the presence of an acute infection.

## 1 Introduction

*Mycoplasma pneumoniae* is known to cause upper and lower respiratory tract infections in humans, particularly in children. Previous studies have indicated that *M. pneumoniae* accounts for 7% to 20% of cases of community-acquired pneumonia among children aged three to fifteen years, with outbreaks occurring globally every three to four years (1). In recent years, the incidence of *mycoplasma pneumoniae* pneumonia among children in China has been on the rise (2). *Mycoplasma pneumoniae* can also result in bronchial remodeling changes, atelectasis, destroyed lung (3), chronic interstitial fibrosis, bronchiolitis obliterans and bronchiectasis (4), as well as extrapulmonary complications such as myocarditis, pericarditis, nephritis, and meningitis (5). Due to the poor immune response and safety issues, there is no effective vaccine for *mycoplasma* infection currently (6). Serological measurement of IgM antibody levels is one of the methods employed for the clinical diagnosis of *M. pneumoniae* infection. Generally, compared to IgG, which indicates a chronic disease or a prior infection, IgM suggests a recent and acute infection (7). Therefore, many researchers utilize a single IgM level, sometimes combined with polymerase chain reaction (PCR), to diagnose acute infection (8–12). Studies have demonstrated that positive IgM antibody results can persist for weeks, months, or even longer after an *M. pneumoniae* infection (13). In China, a large-sample study showed that the age distribution of 70,259 children positive for MP-IgM antibody was as follows: preschool group (43.15%)> school-age group (29.80%)> preschool group (22.92%)> infant group (4.13%) (14). However, there have been rare case reports about persistently positive *M. pneumoniae* IgM antibody titers. Although IgM tests are clinically useful, they can result in misdiagnoses and inappropriate treatment (15). Notably, a positive *M. pneumoniae* IgM antibody test often leads to the misuse of macrolide antibiotics. Recently, the resistance of *M. pneumoniae* to macrolides has continuously increased. It is reported that the region with the highest proportion of macrolide-resistant *Mycoplasma pneumoniae* (MRMP) infection is the Western Pacific region, with a proportion of 53.4% (16). In China, the highest prevalence of MRMP infection was 97.4% in 2019 (17). It is considered an important cause of severe and refractory pneumonia caused by *M. pneumoniae* in some reports (18, 19).

In this study, we aimed to provide a deeper understanding of the clinical importance of positive *M. pneumoniae* IgM antibody titers by conducting a systematic review of data from the multiple hospitalizations of two children who consistently tested positive for IgM antibodies against *M. pneumoniae*.

## 2 Materials and methods

### 2.1 Study subjects

This study retrospectively collected the medical records of two children with persistent positive IgM antibodies against *M. pneumoniae* at our hospital and other hospitals from 2018 to 2023. We compared and serially analyzed pathogen detection data and drug use.

### 2.2 Pathogen detection method

The detection methods used for the etiological tests in this study are described below.

#### 2.2.1 Serologic testing for *Mycoplasma pneumoniae*

IgM antibodies against *M. pneumoniae* were qualitatively measured using the colloidal gold method, following the instructions provided by the manufacturer (Sangon Biotech Co., Ltd., Shanghai, China). The sensitivity and specificity were 97.4% and 100.0% respectively (20). The result was defined as positive when both the detection and control lines displayed color. The result was considered negative when only the control line displayed color, or invalid if only the detection line displayed color. The test was repeated for invalid results.

Quantitative detection of IgM and IgG antibodies against *M. pneumoniae* was conducted using the direct chemiluminescence technique. The kits used in this study were provided by Shenzhen Yahui Long Biological Technology Co., Ltd. (Shenzhen, Guangdong, China). The procedures strictly followed the instructions provided. Samples with IgG concentration >36.0 Au/ml and IgM cutoff index (COI) >1.1 were considered positive.

A specific immune agglutination test (SERODIA-MYCO II Kit; Fujirebio Co., Ltd., Tokyo, Japan) was used to detect trace peripheral serum IgM antibodies against *M. pneumoniae* following the manufacturer's instructions. A positive result was defined as a titer of *M. pneumoniae* antibodies greater than 1:80.

#### 2.2.2 Quantitative polymerase chain reaction assay for the nucleic acids of *Mycoplasma pneumoniae*

Bronchoalveolar lavage fluid (BALF) samples were analyzed using the *M. pneumoniae* nucleic acid quantitative detection kit (PCR fluorescent probe assay; DaAn Gene Co., Ltd., Guangzhou, Guangdong, China), following the instructions provided by the manufacturer. The linear range for quantitative detection is 400–4 × 10^9^ copies/ml. A result greater than 400 copies/ml is defined as positive.

#### 2.2.3 Antigen tests for seven respiratory viruses

Antigens for seven respiratory viruses, including adenovirus, respiratory syncytial virus (RSV), influenza A (InfA); influenza B (InfB), and parainfluenza types 1, 2, and 3, were tested using direct immunofluorescence. Fluorescently labeled monoclonal antibodies were visualized using a fluorescence microscope. The reagents used for this analysis were provided by Shanghai B&C Biological Technology Co., Ltd. (Shanghai, China), and the instructions for the tests were strictly followed.

#### 2.2.4 Multiplex detection of nucleic acids from 13 respiratory pathogens

Various strains of InfA, influenza A H1N1 (InfA H1N1), InfA H3N2, InfB, human parainfluenza, human adenovirus, human bocavirus, human rhinovirus (HRV), human metapneumovirus, human coronavirus (HCoV), human respiratory syncytial virus (HRSV), *Chlamydia pneumoniae*, and *M. pneumoniae* were measured using primers that were included in the nucleic acid detection kit (Ningbo HEALTH Gene Technologies Co., Ltd. Zhejiang, China). The multiplex PCR system and procedures were conducted based on the instructions recommended by the manufacturer.

#### 2.2.5 Next-generation sequencing

Nasopharyngeal swabs and BALF samples were sent to a third-party testing facility for targeted next-generation sequencing (tNGS) (KingMed Diagnostics Group Co., Ltd., Guangzhou, Guangdong, China). In addition, pathogen metagenomic next-generation sequencing (mNGS) was done by Vision Medicals Co., Ltd. (Guangzhou, Guangdong, China).

### 2.3 Statistical analysis

Pathogen detection data and drug use were comprehensively analyzed.

## 3 Results

### 3.1 Case 1

The first case was a girl who was born on August 24, 2017, through a G1P1 term Cesarean section with a birth weight of 2.7 kg. There was no history of familial genetic diseases, and her growth and development were normal. However, the child was hospitalized 11 times due to recurrent pulmonary infections. The relevant pathogenic detection data and drug use information are shown in Table 1.

**Table 1:** Pathogen detection data and drug use information of case 1.

Hospitalization event	Date	MP antibody titer	MP-IgM quantification (COI)	MP-IgG quantification (AU/ml)	Seven viruses	Thirteen pathogens	MP-DNA (BALF) (copies/ml)	tNGS (DNA sequence count)	Use of macrolides
1st	2018-05-25	NA	NA	NA	NA	NA	NA	NA	NO
2nd	2019-11-16	1:80	NA	NA	negative	NA	NA	NA	NO
3rd	2019-11-27	1:80	NA	NA	HRSV	NA	NA	NA	YES
4th	2021-01-20	NA	0.58	<2.00	negative	NA	NA	NA	YES
5th	2021-04-04	NA	0.50	<2.00	negative	NA	NA	NA	NO
6th	2021-10-15	NA	0.38	<2.00	NA	HRV	NA	NA	NO
7th	2022-07-07	NA	0.30	<2.00	NA	negative	NA	BALF: HMPV 33,900, InfA 667, MP 19	YES
8th	2022-07-25	NA	0.27	<2.00	NA	MP, HRV	NA	BALF: MP 61,352	YES
9th	2022-08-10	NA	7.02	>300.00	NA	NA	4.5 × 10^2^	NS: HADV-B 40,816, HCoV-NL63 2084, MP 534	YES
10th	2023-03-12	NA	1.73	>300.00	NA	NA	NA	NS: InfA H1N1 9298	YES
11th	2023-05-23	NA	1.26	>300.00	NA	HRSV	NA	BALF: HRSV-A 56,604, HPIV 51	NO

BALF, bronchoalveolar lavage fluid; NS, nasopharyngeal swab; NA, not available; HRSV, human respiratory syncytial virus; HRV, human rhinovirus; HMPV, human metapneumovirus; InfA, influenza A; InfA H1N1, influenza A H1N1; HADV-B, human adenovirus type B; HPIV, human parainfluenza virus; HCoV-NL63, human coronavirus NL63; MP, *mycoplasma pneumoniae*. Red marks indicate positive MP-IgM.

In this case, we observed a gradual decrease in the serological levels of IgM antibodies against *M. pneumoniae* from August 10, 2022 (COI 7.02) to May 23, 2023 (COI 1.26). Despite having only one mycoplasma infection, the tests for *M. pneumoniae* IgM antibodies remained positive for nearly ten months. In six out of the eleven hospitalizations, macrolide antibiotics were administered.

Medication analysis revealed that the *M. pneumoniae* antibody titer was 1:80 during the third hospitalization, and macrolide antibiotics were prescribed. However, the tests for seven respiratory viruses suggested HRSV infection. During the fourth hospitalization, the *M. pneumoniae* IgM antibody titer revealed a COI of 0.58, and macrolide antibiotics were administered. In the seventh hospitalization, analysis of the BALF indicated that the sequence count of *M. pneumoniae* DNA was 19. In the eighth hospitalization, multiplex nucleic acid tests for 13 respiratory pathogens revealed a positive result for *M. pneumoniae* and the sequence count of *M. pneumoniae* DNA was 61,352 on tNGS. In the ninth hospitalization, the *M. pneumoniae* IgM antibody titer exhibited a 7.02 COI, the BALF *M. pneumoniae* DNA was positive at 4.5 × 10^2^ copies/ml, and the tNGS revealed a sequence count of *M. pneumoniae* DNA of 534. During the 10th hospitalization, the *M. pneumoniae* IgM antibody titer was 1.73 COI, and macrolide antibiotics were administered. Although tNGS detected InfA H1N1 but not *M. pneumoniae*.

### 3.2 Case 2

Case 2 was a girl who was born on February 12, 2016. She was the firstborn of his parents delivered at full term. She weighed 3.45 kg at birth. She had no history of familial genetic diseases, and her growth and development were normal. The child was hospitalized six times due to medical problems, such as plastic bronchitis, bronchial occlusion, and atelectasis secondary to mycoplasma infection. Table 2 shows pathogen detection data and medication information.

**Table 2:** Pathogen detection data and drug use information of case 2.

Hospitalization event	Date	MP-IgM qualification	MP-IgM quantification (COI)	MP-IgG quantification (AU/ml)	Thirteen pathogens	mNGS (DNA sequence count)	tNGS (DNA sequence count)	Use of macrolides
1st	2022-02-21	positive	NA	NA	Negative	NA	NA	YES
2nd	2022-03-05	positive	NA	NA	Negative	NA	NA	YES
3rd	2022-03-29	NA	6.33	>300.00	Negative	BALF: Hin 15,998, HCMV 11	NA	YES
4th	2022-04-12	NA	5.58	>300.00	NA	NA	NA	NO
5th	2022-10-23	NA	3.19	>300.00	HRV	NA	NA	YES
6th	2023-05-30	NA	2.44	>300.00	NA	NA	BALF: HCoV-OC43 16,392, SPn 2,672, Hin 2,326	YES

NA, not available; BALF, bronchoalveolar lavage fluid; Hin, *Haemophilus influenzae*; HCMV, human cytomegalovirus; HCoV-OC43, human coronavirus-OC43; SPn, *Streptococcus pneumoniae*.

Red marks indicate positive MP-IgM.

In case 2, the qualitative serological tests for *M. pneumoniae* IgM antibodies showed positive results from February 21, 2022, to May 30, 2023 (i.e., more than 15 months). The *M. pneumoniae* IgM antibody titers gradually decreased from a COI of 6.33 on March 29, 2022, to a COI of 2.44 on May 30, 2023. These findings indicated a single *M. pneumoniae* infection. During the six hospitalizations, macrolide antibiotics were prescribed five times.

The reason for the first and second hospitalizations was a recurring condition. The child was readmitted with a fever five days after the first hospitalization. During the second hospitalization, the pulmonary CT scan revealed infections in the right middle lobe and the left lingual lobe, accompanied by localized atelectasis in the right middle lobe. Moreover, the lesion area had expanded compared to the previous pulmonary CT findings during the first hospitalization. Qualitative tests for *M. pneumoniae* IgM antibodies were positive on both occasions, suggesting a mycoplasma infection. In the third hospitalization, the *M. pneumoniae* IgM antibody titer exhibited a COI of 6.33. The BALF mNGS was positive for *Haemophilus influenzae* (Hin) and human cytomegalovirus (HCMV) and did not detect mycoplasma. In the fifth hospitalization, the *M. pneumoniae* IgM antibody titer had a COI of 3.19, and the tests for 13 pathogens detected HRV infection. During the sixth hospitalization, the *M. pneumoniae* IgM antibody titer exhibited a COI of 2.44. tNGS was positive for HCoV-OC43, *Streptococcus pneumoniae*, and Hin, but negative for mycoplasma.

## 4 Discussion

### 4.1 Dureation of *M. Pneumoniae* IgM positivity in children

In this study, two children underwent multiple serological tests for *M. pneumoniae*. One patient had prolonged serological positivity for *M. pneumoniae* IgM antibodies for nearly ten months, whereas the other showed persistent positivity for over fifteen months. After *M. pneumoniae* infection, the body can produce specific IgM, IgG, and IgA class antibodies. Previous studies have shown that IgM antibodies usually emerge one week after an *M. pneumoniae* infection, peak at two to three weeks, decrease at four weeks (21–23), and may persist in low levels for months or even years (24). In clinical practice, most children who were positive for *M. pneumoniae* IgM antibodies after effective treatment had generally no need for long-term follow-up examinations. Therefore, patients with persistent positivity for IgM antibodies against *M. pneumoniae* have rarely been reported.

### 4.2 Conventional understanding of *M. Pneumoniae* IgM positivity

IgM antibodies are primarily considered indicators of acute inflammation (25–27), appearing early during the host immune response to pathogens and showing decreased levels shortly after the appearance of IgG antibodies (28). IgM antibodies against *M. pneumoniae* are generally believed to be a diagnostic indicator for early infection (22, 29–31). Moreover, some researchers have reported that among patients with cough, fever, and expectoration, positive results for *M. pneumoniae* IgM antibodies can be used to diagnose recent *M. pneumoniae* pneumonia necessitating antibiotics (32). However, the use of macrolide antibiotics based solely on a positive IgM test as an indicator of a recent infection can increase the risk of drug misuse. Furthermore, many cases of refractory *M. pneumoniae* pneumonia have been recently reported among children (33–35).

### 4.3 Persistent *M. Pneumoniae* IgM positivity does not imply ongoing infection

In this study, detecting IgM antibodies against *M. pneumoniae* was the basis for prescribing macrolide antibiotics in 6 out of 11 hospitalizations of case 1 and 5 out of 6 hospitalizations of case 2. As can be seen in the two tables, the values obtained using the MP-IgM quantitative detection of the two patients gradually decreased. During the ninth hospitalization, case 1 tested positive for MP-IgM and DNA, which was the primary infection. In the tenth and eleventh hospitalizations, the patient was positive for MP-IgM, but the tNGS test was negative for MP while positive for InfA H1N1 and HRSV-A, which did not support persistent or active *M. pneumoniae* infection. Case 2 was positive for MP-IgM during the third to sixth hospitalizations, but the DNA test was negative and did not support persistent or active infection.

### 4.4 Positivity of *M. Pneumoniae* IgM antibodies and the risk of macrolide misuse

For case 1, using macrolide antibiotics in the seventh, eighth, and ninth hospitalizations was deemed appropriate based on the positive test results for *M. pneumoniae* DNA and IgM antibody. However, the use of macrolides was unreasonable during the third, fourth, and tenth hospitalizations based on the low *M. pneumoniae* antibody titer, decreased levels of *M. pneumoniae* IgM antibody titers, and the presence of other etiological results. For case 2, using macrolide antibiotics during the first and second hospitalizations was appropriate based on the positive tests for *M. pneumoniae* IgM antibody and other negative etiological results. However, during the third, fifth, and sixth hospitalizations, the use of macrolides was deemed inappropriate based on the positive *M. pneumoniae* IgM and IgG antibody titers because *M. pneumoniae* DNA result was negative and other etiological factors were present. Upon analysis, the excessive use of macrolides was problematic for both cases.

Based on these results and other similar cases (36–39), the use of macrolide antibiotics based on positive IgM antibody titers against *M. pneumoniae* has likely contributed to the development of *M. pneumoniae* resistance. In fact, the excessive use of macrolide antibiotics has been identified as an important factor contributing to the rising global prevalence of MRMP cases (1, 40), which is a serious issue that requires attention. Moreover, it is reported that azithromycin can also lead to gut microbiota imbalance (41, 42).

### 4.5 Reconceptualizing the clinical relevance of *M. pneumoniae* IgM antibodies

This study underscored the importance of dynamic monitoring of *M. pneumoniae* IgM antibody titers to ascertain the presence of an acute mycoplasma infection. Comparing the antibody levels before and after an infection is essential for this purpose. A rapid increase in *M. pneumoniae* IgM antibody titers within a short term is typically indicative of an acute infection. In these two cases, the IgM antibody titers against *M. pneumoniae* consistently decreased. Thus, observing the antibody titer trend, in conjunction with data obtained for other etiological factors, can provide a more comprehensive understanding and help distinguish acute infections from other conditions. Many researchers have emphasized the reliability of employing two short-term evaluations of serological IgM titers to screen patients for acute infection and minimize the selection bias encountered in studies on *M. pneumoniae* (43–45). Guidelines or protocols should pay more attention to standardize the interpretation of IgM antibody titers in clinical practice.

Although several studies and guidelines (31, 44, 46, 47) have indicated that IgM antibodies against *M. pneumoniae* may persist for an extended period, the specific duration has yet to be determined. Despite the demonstrated value of IgM level in numerous cases, many clinicians and laboratory personnel lack awareness about the importance of *M. pneumoniae* IgM antibodies in diagnosing acute or recent infections. This study highlighted the possibility of persistent positive IgM antibody titers against *M. pneumoniae* for more than one year and indicated that a positive *Mycoplasma pneumoniae* IgM test does not necessarily indicate the presence of an acute infection. Furthermore, these results indicated that macrolide antibiotics should not be misused solely based on *M. pneumonia* IgM test.

Studies on different Etiologies have also shown that specific IgM antibodies do not always indicate acute infection. Lassa virus-specific IgM serostatus cannot be considered a diagnostic marker of acute infection (48). The presence of *Toxoplasma gondii*-specific IgM antibodies also does not necessarily suggest an acute infection (49). Furthermore, it has been reported that specific IgM antibodies against parvovirus B19 infection in pregnant women can be persistently detected for up to nine months (50). A prospective observational study revealed that Zika virus IgM antibodies can persist for 237.7 days (128.7–459.5) following the estimated time of detectable infection via plasma nucleic acid amplification testing (51). West Nile virus(WNV) IgM antibodies can persist for more than three years in 12% of patients with WNV infection (52). A study described the persistence of IgM against SARS-CoV-2 for up to one year, so the authors believed that the use of IgM antibodies to identify the infection stage needs to be evaluated with caution (53).

### 4.6 Limitations

The main limitations of this study were the small number of cases and retrospective data analysis. While, in clinical practice, most children who were positive for *M. pneumoniae* IgM antibodies after effective treatment had generally no need for long-term follow-up examinations. Therefore, patients with persistent positivity for IgM antibodies against *M. pneumoniae* have rarely been reported. Further studies with long-term monitoring of larger sample sizes are needed to confirm these findings. Additionally, it is critical to consider whether the current positive cut-off value for the *M. pneumoniae* IgM antibody test is appropriate.

## 5 Conclusions

Positive IgM antibody titers for *M. pneumoniae* can persist for an extended period and even last over one year. Thus, positive *M. pneumoniae* IgM levels do not necessarily suggest a recent infection. Therefore, it is esential to highlight the implications of IgM antibody for clinical decision-making and the need for further research to establish standardized criteria for interpreting IgM antibody titers. When considering the use of macrolide antibiotics, it is crucial to not rely solely on *M. pneumoniae* IgM levels to ascertain the presence of a recent infection. Instead, a comprehensive approach should be taken by comparing the trend of *M. pneumoniae* IgM titers and dynamically analyzing the data. Furthermore, the results of other detection methods for relevant etiological factors should be considered to ascertain an accurate diagnosis and comprehensively confirm the presence of *M. pneumoniae*.

## Data Availability

The original contributions presented in the study are included in the article/Supplementary Material, further inquiries can be directed to the corresponding author.
